# Does urbanization make emergence of zoonosis more likely? Evidence, myths and gaps

**DOI:** 10.1177/0956247819866124

**Published:** 2019-09-14

**Authors:** Sohel Ahmed, Julio D Dávila, Adriana Allen, Mordechai (MUKI) Haklay, Cecilia Tacoli, Eric M Fèvre

**Keywords:** health hazards, livestock, risk accumulation, urban global South, urbanization, zoonosis

## Abstract

Rapid urbanization in the global South is adding epidemiological and nutritional challenges and increasing disease and health burdens for citizens. Greater movement of people, animals, food and trade often provides favourable grounds for the emergence of infectious diseases, including zoonoses. We conduct a rapid evidence scan to explore what is known and hypothesized about the links between urbanization and zoonosis emergence. This points to rapid demographic growth, migration and density, increased movement of people and animals, and changes in land uses as the main processes linked to the prevalence of zoonosis in the urban global South. We argue that this emerging global health challenge is also deeply connected with the urbanization of poverty and inequalities within cities. Tackling the micro-level causal relationships between urbanization and zoonosis requires urgent attention to living conditions, as well as the wider socioenvironmental transitions and structural drivers that produce and reproduce risk accumulation in urban settings.

**Figure:**
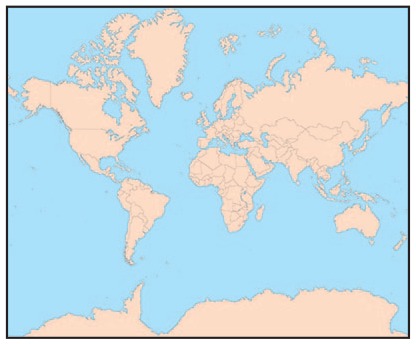


## I. Background and Rationale

The publication of *Urban Health in Developing Countries* by Trudy Harpham and Marcel Tanner in 1995^(1)^ triggered a new generation of scholarship acknowledging the crucial relationship among urbanization, poverty and health in the global South.^(2)^ Into the 21st century, increasing urbanization and inequality, coupled in some regions with political instability and humanitarian crises, bring to the fore the urgent need to reappraise how urban health is understood and tackled as a global challenge. This has also been recognized by Sustainable Development Goal (SDG) 11, to “Make cities and human settlements inclusive, safe, resilient and sustainable”. Part of the urban health agenda has to be a broader understanding of the risks of infectious diseases; as argued by Vojnovic et al., *“growing global interconnections and the speed of travel have transformed the spread of (and speed of spread of) infectious disease and related epidemic control”*.^(3)^ The emergence and reemergence of zoonotic diseases^(4)^ relate to the multi-scalar and interrelated drivers that make cities both producers of health vulnerabilities and enablers of improved health. The aims of this paper are to examine what is known and assumed about such drivers, and to identify what actual and potential explanations have been sidelined and therefore require greater attention from scholars and policymakers.

Globally, the share of the population living in urban areas has been rising rapidly since the second half of the last century: while 30 per cent of the world’s population was urban in 1950, this share rose to 55 per cent in 2018. By 2050, the urban share of the world’s population is projected to increase to 65 per cent, with almost 90 per cent of this growth taking place in Asia and Africa.^(5)^ Thus, it is not surprising that municipal governments across these regions are struggling to keep pace with the infrastructural improvements and protective measures required, while allocating resources more equitably across all social groups.^(6)^ These trends are compounded by unprecedented challenges in urban food security, resulting from global changes in food production and consumption systems.^(7)^ In many urban centres in the global South, there is higher demand for meat, dairy products and more highly processed foods.^(8)^ These changing dietary practices have prompted the so-called “livestock revolution”.^(9)^ However, as argued by Sumberg and Thompson,^(10)^ this “revolution” has to be reconsidered in light of evolving perspectives and contemporary trends in livestock production and consumption. From 1980 to 2004, meat production doubled, and is set to double again by 2020.^(11)^ Demand for livestock products is predicted to double from 200 to 400 kilocalories per person per day in sub-Saharan Africa and South Asia between 2000 and 2050.^(12)^ Traditional practices of domesticating animals that started approximately 10,000 years ago have been gradually replaced with intensive industrialized systems, particularly in Asia, Africa and South America.^(13)^ This is changing the way food systems work globally, including in urban areas.

When compounded with rapid urban demographic growth, these trends are bringing additional health challenges mainly to the urban poor, who increasingly face a quadruple disease burden of infectious and chronic diseases, and mal- and over-nutrition.^(14)^ Various studies in China, East and Southeast Asia, and Africa recognize urbanization drivers and outcomes – such as environmental degradation linked to air and water pollution, higher population density, rural–urban migration, inadequate healthcare provision and changes in peri-urban land uses – as closely associated with many infectious and communicable diseases.^(15)^ Many non-communicable diseases, mainly cardiovascular diseases, diabetes, cancers and chronic respiratory diseases, are also strongly correlated with the above-mentioned trends. They are linked as well with nutritional transitions, polluted air and water, and a lack of green walking and cycling spaces.^(16)^

With demographic and nutritional transitions occurring for various reasons, many regions are also experiencing encroachment of wilderness areas and rapid urban land cover expansion at a rate that exceeds by three times the average global rate at which national populations become urban.^(17)^ Intense movements of people and foodstuffs, including animal products, are rapidly changing environmental conditions and also generating favourable grounds for the (re)emergence of infectious disease vectors with increasing epidemiological complexities.

These changes suggest the need to critically examine the link between the forces underpinning rapid urban growth and the emergence of zoonotic diseases. Zoonotic diseases are, according to Schelling and colleagues, *“transmitted via food products such as meat and dairy products and other animal products, water and waste”*.^(18)^ In fact, it is claimed that most infectious food-borne diseases are zoonotic in nature.^(19)^ Out of more than 1,400 human pathogens reviewed by Taylor, Latham and Woolhouse^(20)^ and Woolhouse and Gowtage-Sequeria,^(21)^ more than half are considered zoonotic. Some zoonoses are called emerging or reemerging^(22)^ because of their higher incidence in the last two decades, together with increasing geographical coverage and their potential to spread more rapidly and widely in the near future. The World Health Organization (WHO) estimates that 61 per cent of all human diseases are zoonotic in origin, while 75 per cent of new diseases discovered in the last decade are zoonotic. A recent study claims that 26 per cent of the infectious disease burden (measured in terms of disability-adjusted life years, or DALYs^(23)^) in developing countries arises from zoonoses, compared with a mere 0.7 per cent in developed nations. However, the first figure is likely to be an underestimation, resulting from heavy under-reporting and misdiagnosis.^(24)^ According to Havelaar et. al.,^(25)^ diarrhoeal diseases are amongst the most common diseases in the global South, half of which have zoonotic origins. Yet there is no particular study that examines the key drivers underpinning the relation between what drives urbanization (which can be understood both as increases in the share of the urban population of a country and as the physical expansion of cities) and zoonosis in the urban global South.

We have looked into scholarship to explore what drivers of urbanization are predominantly hypothesized and are related to the increased prevalence of selected zoonosis in urban centres. In the next section, we explain the methods adopted for our evidence scan review. In the third section, we examine the dominant knowledge narratives that emerge from the selected peer-reviewed scholarship. In the fourth section, we reflect critically on these findings and explore neglected narratives and drivers that require more attention in future research. We conclude with some remarks about the transformative possibilities of such knowledge.

## II. Methodology

**Figure 1:**
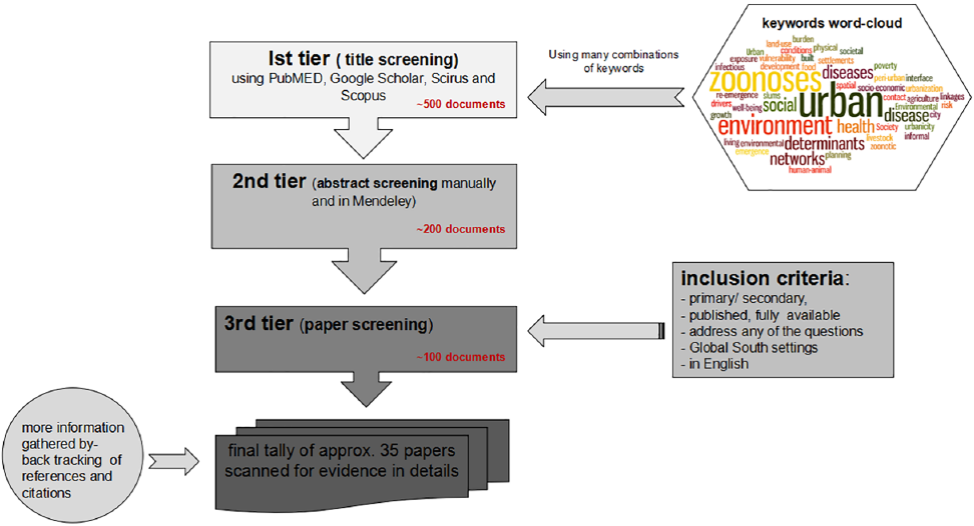
Methodology followed in reviewing the literature

The study underpinning this article focused primarily on scanning scholarly evidence and narratives on major food-borne and livestock-borne direct zoonosis that are emerging or reemerging in the urban global South. The sources reviewed include research articles and empirical reports, including grey material. After consulting epidemiologists, we initially searched research databases such as PubMed, Google Scholar, Scirus and Scopus using 40 keywords^(26)^ (Figure 1). Upon scanning some of the key literature, a further search for particular zoonosis-specific evidence was made, both by using keywords and by searching manually, including back-tracking and citation tracking of related articles, reports and books. The websites of relevant institutes or international agencies were also explored for reports, data, policies and evidence. The following inclusion criteria were used: studies had to address the key question behind this scan, and be situated in the context of the urban global South; be primary research or reviews; be published in peer-reviewed outlets; be readily available online, in print or from relevant organizations; and be available in English in their abstract, journal article, or report form (Figure 1). As shown in the figure, from initial title screening of approx. 500 documents, only 35 documents were selected for the evidence scan after abstract and further paper screening using the criteria above.

## III. Urbanization, Health and Zoonosis: Evidence and Assumptions

In a 2005 systematic review, Woolhouse and Gowtage-Sequeria^(27)^ found that changes in land use or agricultural practices were the primary drivers in the emergence or reemergence of zoonotic diseases. Other important drivers identified included: demographic change, poor population health, pathogen evolution, contamination of food and water, international travel and trade, failure of public health programmes and climate change. However, the authors did not look into whether the reviewed trends and correlations differed between urban and rural areas. A more recent study by Mackenstedt et al. argues that the *“role of potential hosts for transmission of a zoonotic disease in urban or peri-urban areas cannot be extrapolated from data obtained in rural areas”*.^(28)^ Allen et al.^(29)^ argue that our understanding of the demographic, environmental and locational factors underlying the emergence of zoonoses remains rudimentary. What follows from these observations is that the multiple drivers underpinning the transmission of zoonotic pathogens in urban areas are still poorly understood, particularly in the urban global South.

In the following subsections we examine the dominant narratives found through the bulk of the scholarship reviewed concerning what is known, hypothesized or assumed about the role of rapid urban growth as a driver behind the emergence, incidence and persistence of zoonotic diseases in the urban global South.

### a. “Urban advantage” as a myth

The belief that health conditions are better in urban than rural areas because of faster economic growth is often misguided, resulting from the overemphasis on city-scale aggregate data that tends to disguise intra-city differences among groups of urban dwellers.^(30)^ In reality, the so-called “urban advantage” that cities can offer residents and recent migrants, which is due to their higher concentration of health services and income-earning opportunities, can turn into an “urban penalty” for certain socioeconomic groups, particularly in the urban global South. For instance, when the availability of data allows groups of low-income residents to be singled out, it has been shown that the incidence of both disability or morbidity arising from malnutrition, and of child mortality related to respiratory and water-borne illness through faecal–oral routes, tends to be higher in some urban neighbourhoods than in rural areas.^(31)^ A key factor in helping reduce this “urban penalty”, by improving health conditions and reducing the gap between wealthier and poorer areas in cities, is the availability of reliable basic infrastructure services (clean water, sanitation, well-maintained roads), health services and education. Local governments often bear responsibility for these services.^(32)^

### b. Intra-city socioenvironmental inequalities

The failure of the so-called urban advantage to deliver for many people in the urban global South is especially pronounced in many African cities. Here, a high proportion of the urban population lives in “slums” or informal settlements – with shares in countries like Sudan and Central African Republic reaching as much as 94 per cent.^(33)^ Large socioeconomic disparities can also be seen in inadequate access to basic infrastructure services by large proportions of the population; for example, in 2015 two-thirds of the urban population in the least developed countries lacked access to water piped on premises, and 53 per cent lacked access to “improved” sanitation.^(34)^

Unsafe water and inadequate sanitation are two of the prime reasons for the high prevalence of diarrhoeal diseases in cities.^(35)^ Open drainage and proximity to refuse dumping sites are often problems for poorer communities, resulting in higher prevalence of rodent and parasite-borne diseases. For instance, there is significant household clustering of the Leptospira infection in the informal settlements of Salvador, Brazil and Tamil Nadu, India. This is claimed to be due to increased exposure to sources of environmental contamination, such as the presence of open sewers,^(36)^ particularly in flood-risk areas and areas near refuse dumps.^(37)^ Similar drivers are also mentioned for Patna, India^(38)^ and for Banda, Uganda.^(39)^ Katukiza et al.^(40)^ looked into the magnitude of microbial presence through different exposure pathways for water-borne disease in the Bwaise III informal settlement in Kampala, Uganda. Surface water in open drainage channels holds the highest pathogen burden (39 per cent), followed by greywater in tertiary drains (24 per cent) and storage containers (22 per cent), while tap water holds a very low percentage (0.02 per cent).^(41)^ The authors estimate that the exposure to different bacteria and viruses costs the Bwaise III population around 680 disability-adjusted life years (DALYs) per 1,000 persons per year. (This is much higher than the WHO reference level of tolerable risk measured in DALYs.) E. coli O157:H7 causes the highest estimated share of the infections, followed by rotavirus and salmonella.

### c. Rural-to-urban migration

It is also argued that high rural-to-urban migration can increase the risk of zoonoses. According to Alirol et al.,^(42)^ this can arise in three ways:

- Rural migrants are accompanied by pathogens due to interaction with rural wildlife or livestock.
- If migrants are already infected before settling in a city, this exposes the existing urban populace to diseases (e.g. in Kinshasa, Democratic Republic of Congo, the 10-fold increase in the incidence of African trypanosomiasis is believed to be attributable to migration).
- Migrants themselves can fall victim to endemic diseases common to places they move into. This was reported in Kabul, Afghanistan, where the presence of migrants contributed to the reemergence of leishmaniasis (a non-food-borne zoonotic disease), which had been dormant amongst local people who were immune to it.^(43)^

Apart from such risk assumptions attached to migration, high rural-to-urban and circular migration can lead to more intensified urban–rural linkages,^(44)^ which can result in more diverse and intense contact with animals. Animals brought to cities through rural–urban migration may have a key role in sparking outbreaks or seeding new genetic strains from rural spaces in high-density urban settings.

### d. Industrialization and trade of animal and animal-centred food products

Several scholars point to increased movement of people, animals and animal-centred food products at multiple scales. With rapid nutritional transitions across many cities in the global South, there is an increased demand for animal products, thus intensifying the trade and transport of live animals and animal products within cities and across national borders. This has ramifications for industrial-scale animal production near or within cities. This is particularly in the case of monogastric species, such as poultry and pigs, because of the ease of intensification and higher reproduction rates, which in turn enable increased rates of animal-to-human contact along high traffic corridors.^(45)^ This emerging nexus in some major cities, termed “peri-urbanization of industrial animal agriculture”, has the potential to be a major entry point and transmission route for zoonoses.^(46)^ International value chains also have a role to play, as shown from the outbreak of avian influenza in Southeast Asia in 2004. This was identified as having started in Lhasa, Tibet, when 5,000 live chickens were being transported to Lanzhou, China, some 1,600 kilometres away.^(47)^ Similarly, Rift Valley Fever (RVF) was introduced to Yemen when infected animals in large numbers were traded from the RVF-endemic Horn of Africa.^(48)^ Even though, due to a number of factors that tend to reinforce each other, poor people are more prone to being victims of diseases that stem and propagate from such movements, these can also spread to other groups in the city. Intensive farming, trade, transport and complex value chains within cities add to the complexity of the problem. In 2006/2007, disease outbreaks were reported in 29 out of 69 districts in Kenya, mostly concentrated in Garissa, Baringo and Kilifi. In Baringo, disease outbreaks can be linked to animal-to-human transmission, since most of the human cases occurred close to livestock.^(49)^ Increased urban demand for food creates a high degree of crossover between formal and informal food systems,^(50)^ resulting in disease risks to all members of the population regardless of their socioeconomic grouping. Despite increased urban food supply, the poor have less choice in their food sourcing and are at greatest risk of disease.^(51)^

### e. Land-use changes

As mentioned earlier, Woolhouse and Gowtage-Sequeria^(52)^ considered changes in land-use and agricultural practices to be the main drivers behind the emergence of 177 human pathogens. Most urban centres across the global South experience unplanned and uncontrolled urban growth related to the absence of land-use planning and strategic planning frameworks, or, where they are present, the inability to adhere to them, as a result of pressure from vested interests.^(53)^ Lack of planning and adequate management in converting agricultural and other non-urban land to urban use^(54)^ has several negative externalities. First, building on flood-prone land that has been drained disturbs the ecosystem. This results in, for example, prolonged waterlogging after heavy rainfall, which heightens the risk of water-borne infectious diseases.

Second, encroachment on the natural ecosystem and wildlife by agricultural and urban land uses will expose humans and their domestic animals to areas with higher risks and a wider range of vectors. For example, habitat destruction and fragmentation in Cambodia, Thailand, India, Bangladesh and Madagascar brought fruit bats closer to humans and domestic animals, causing outbreaks of Nipah virus infection.^(55)^ Based on a recent (1973–2010) and historical (1788–1973) review of infectious disease literature of humans and animals, McFarlane et al.^(56)^ found that 22 per cent of the reviewed emerging infectious diseases are associated with land use and land cover change. Most frequently, natural landscapes have been removed or replaced with agriculture, plantations, livestock or urban development. Historically, clustering of vector-borne, zoonotic and environmental disease emergence also follows major periods of extensive land clearing.^(57)^

Recent research on the links between land use and antimicrobial resistance (AMR)^(58)^ in E. coli in Nairobi offers novel insights into the issue. There is an ecological gradation in patterns of land use in Nairobi, with wealthier and less densely populated neighbourhoods tending to be more ecologically diverse than poorer, more densely populated areas. The data show that there is also a gradation in AMR along this axis: while there is more diversity of AMR genes but less virulence in the highly dense populations, there is less AMR diversity but more virulence in the more ecologically diverse, richer neighbourhoods.^(59)^

## IV. Reflection on the Evidence Scan: Focus on Marginal Narratives

Following the previous section, the most frequently cited urbanization drivers in relation to zoonoses can be summarized as: urbanization in the forms of rapid urban growth and increased density of land occupation; heightened movement of people, animal and animal-sourced products; rural-to-urban migration; intra-city inequalities; and changes in land use. Yet in the following sub-sections we argue that overreliance on certain drivers can lead to simplification, which can cause the bigger picture to be missed. We discuss the more marginalized narratives, or evidence and arguments that have received limited or no attention in the zoonosis and urbanization scholarship.

### a. Interaction of zoonosis with urbanization drivers is a “wicked problem” operating in a complex system

Zoonosis itself is a “complex problem”, as the many drivers that are likely to influence the emergence, persistence and/or prevalence of zoonotic diseases are interconnected, multi-level and multi-scalar. In most of the studies reviewed, scholars recognize zoonosis as a complex problem but then base their analysis mostly on quantitative probabilistic models that restrict the presentation of results, outcomes and likelihoods, hindering it from embracing the full complexity and diversity of conditions in which urbanization and zoonosis are interrelated.^(60)^ Also common in the literature are references to the control and prevention of zoonosis as a “wicked problem”, meaning that we lack both a clear definition of the problem and the capacity to solve it.^(61)^

We looked into a few studies that explored the relation of urbanization drivers to non-communicable diseases, and found that scholars in general recognized that many of these drivers are crosscutting and could be placed on a continuum. This highlights Bai et al.’s observation that *“pathways through which urbanisation affects health are complicated and multifactorial”*,^(62)^ as interactions of humans with their urban environments are *“multi-faceted, diverse, dynamic, complex and evolving”*.^(63)^ Drawing on Gatrell’s take on salient features of complex systems for health,^(64)^ we can gather that the relations inherent in urbanization or city life have many features reminiscent of classic complex systems. But Gatrell’s notion of complex systems is different from the classic system-based approach, as he emphasizes explanation and understanding rather than prediction and control.^(65)^ The use of such a lens allows for a critical reading and understanding of the wider context, which is not reductionist or positivist in nature; nor is it a simple, linear system-based approach. This should be the case for zoonoses, given their reliance on complex urban transitions and the food–animal–human–environment nexus.

For zoonoses, the risk of disease emergence is also “emergent” and “uncertain” (e.g. meat-handling behaviour, along with practices at slaughterhouses and by consumers). Yet those uncertainties are mostly treated as individual behaviours, ignoring system-wide implications that explain why they cannot be “summed” or “averaged” to know or predict system-wide behaviour. Similarly, the collective health outcomes of a neighbourhood cannot be summed up from individual outcomes, as this misses the importance of spatio-temporal, multi-scale interactions within the food–animal–human–environment nexus. Again, such complex systems with non-linear feedback loops can have butterfly effects – such as when a “blip” of pathogen overload leads to disease outbreak with differential implications for different people. Hard-to-diagnose diseases like those of zoonotic origin are more likely to pose higher risks to those living in informal settlements, many of which host systemic pathogenic environments manifested in higher burdens of multiple diseases and poorer health profiles when compared with the rest of the city, making them perfect breeding grounds for zoonoses and other infectious disease outbreaks. Yet we are not learning much from the horrific lessons of the Ebola epidemic in West Africa, and the risk of infection for poorer communities by many other zoonoses remains significantly neglected in the urban global South.

### b. A techno-scientific approach dominates knowledge production and policy narratives

The majority of studies reviewed in this particular field rely on realist or techno-scientific (statistical or modelling) approaches for knowledge generation and focus on particular zoonotic diseases without considering their context. This in turn often leads to certain knowledge generation pathways that can only produce apolitical knowledge outcomes: those that can be measured independently of wider socioeconomic processes. Hence, it is reasonable to conclude that the literature and evidence we looked into often ignore underlying social, economic and environmental forces associated with urbanization in the global South. With a few exceptions,^(66)^ overreliance on a scientific, apolitical view of zoonotic emergence and the generation of policy narratives can be problematic. As highlighted by Craddock and Hinchliffe^(67)^:

“Who gets sick and where, are not simply ecological or demographic outcomes. Often forces of a political and economic nature create disease, and more crucially, determine the manner of its management and control. Development interventions can displace people to marginal places, making them vulnerable to disease.”

Many of the outbreak narratives driven by scholarship from the global North focus on the consequences and not causes of ill health. Neglected causes are in many cases political and economic factors that generate the accumulation of disease risk and vulnerabilities. These factors are often linked to historical patterns of underdevelopment, which perpetuate poorer health outcomes for the urban poor. The recent Ebola outbreak in the Sierra Leone–Liberia–Guinea border region is a good example that demonstrates the importance of political economy perspectives in understanding the drivers of health risk accumulation. As put by Dzingirai et al.^(68)^:

“[Post-colonial] development pathways have fostered inequality and failed to address corruption or elite capture of resources, combined with a systematic under-investment in state institutions precluding the establishment of resilient health systems, livelihoods and living conditions. . .[Even] before Ebola, many people decided against formal healthcare facilities, favouring instead traditional healers and informal vendors with their more personal approach and pluralistic understandings of disease and therapy. . .[A] mix of conflict and limited opportunities in rural agriculture has seen the capitals of Liberia and Sierra Leone grow rapidly. Poorly planned, with limited sanitation and lacking essential services, these dense urban areas proved fertile ground for the virus. . .[Neglected] health services combined with growing gaps between the elites and governments who followed non-inclusive development strategies, and the rural and urban poor whose trust and livelihoods are undermined by them, has compromised responses to outbreaks. . .[Rumours] that Ebola was manufactured to make money, or kill people, provoked widespread fear, violence and avoidance. The plausibility of these rumours is rooted in people’s experiences of acutely unequal and deadly political economics, from slavery to modern day corruption, where extraordinary wealth has been generated for some at the expense of others.”

Similar long-term conditions or events, such as war and previous or historical conflicts and the continuity of colonial legacies, affect the right to health of a vast majority of urban dwellers. Africa has been the epicentre of over 100 armed conflicts between 1989 and 2000, with an estimated 40 million people displaced during this period and becoming vulnerable to deadly diseases such as Ebola and meningitis.^(69)^

### c. Informality or informal and cultural practices are often blamed as a prime driver of zoonoses

Informality as a driver of the (re)emergence of zoonoses has seldom been explored in health scholarship, yet it is common among policymakers to blame and condemn informal practices, for instance by forbidding the sale of livestock and products in informal settlements and markets. We found a few cases that shed light on the sale of animal-sourced foods or products in informal markets, the domestic practices adopted and the subsequent health outcomes.^(70)^ Informal practices of live/wet markets for cattle and poultry are deeply linked with meat processing industries in many cities of the global South. Many livestock products are sold in informal markets, where food safety regulations are poorly implemented, with traditional processing and retailing practices dominating over official guidelines and standards. Sufficient infrastructure services (roads, water, sanitation) and storage (refrigeration) options are lacking, while not much assistance from the state or the non-governmental sector is available.^(71)^ These informal markets are also highly varied in nature, yet self-organized with complex systems of governance and control. The dominant narrative amongst city regulators and policymakers is that these are places of high risk to exposure to zoonotic diseases, and with low food quality resulting from all these factors and conditions. For example, moderate to high prevalence of campylobacteriosis has been identified in Morogoro, Tanzania, affecting both humans and poultry; poor hygiene and management practices associated with an intensive production system were argued to be responsible.^(72)^

Yet studies from East Africa, Northeast India and Vietnam show, according to Grace, that *“food sold in formal markets, though commonly perceived to be safer, may have lower compliance with standards than informally marketed food”*.^(73)^ While draconian measures are often enforced to govern the system, policymakers and city managers often overlook the fact that informal markets are and will remain the main source of animal-sourced food in Africa and Asia. Furthermore, they offer fresh, local produce from local breeds, cheaper than that in formal outlets. They also provide lifelines to local communities that are tightly linked with such small enterprises – simply based on trust, credit and other social network services.^(74)^

Likewise, although pathogens like E. coli, salmonella and cryptosporidium contaminate meat and milk,^(75)^ perceptions of zoonotic risk are often misguided. For example, some studies in the settlement of Dagoretti in Nairobi (Kenya) found that the risk of cryptosporidium was higher for vegetable consumption than for the consumption of meat and milk.^(76)^ Consequently, out of fear or misconceptions, top-down campaigns against the wrong targets, often adopted during disease outbreaks, drive consumers to stop buying products from the informal traders, which seriously threatens the livelihoods of these marginalized small enterprises.^(77)^

Dominant traditional or cultural practices like consumption of raw milk, closer contact with animals near food production and consumption areas, and poor food hygiene are often considered responsible for the prevalence of any zoonotic disease. But high exposure does not always imply disease, if the hazard is managed. For example, Grace argues that it is often ignored that *“in urban East Africa. . .almost all consumers boil their milk before drinking it, the presence of germs in milk presents little risk”*.^(78)^ Furthermore, human mobility patterns within urban centres and across urban and rural areas determine divergent epidemic dynamics and pathways.^(79)^

### d. Most practices and evidence regarding disease prevalence are often understood as being gender neutral

Scholarship examining zoonotic diseases from a gender perspective is still rare. A more critical lens is required to understand women’s and men’s roles, rights (access to and control of resources), division of labour, interests and needs. In particular, there is a need for more analysis of how gender roles affect exposure to hazards, as well as the capacity to reduce, prevent and manage risk.^(80)^

Yet we found a few exceptions in recent scholarship. In a study in Nairobi, Kenya, significant gender differences were observed for cases with cryptosporidiosis.^(81)^ Along with farm workers, women at home are more exposed to certain pathogens as a result of caring for cattle and handling raw milk, but they are also more knowledgeable about cryptosporidiosis and risk mitigation practices than men, irrespective of socioeconomic differences.^(82)^ In another study in Ibadan, Nigeria, gender differences came out as a significant marker of improved food safety practices, as butchers’ associations with more women had better safety practices, better quality of meat, and less gastro-intestinal illness amongst consumers.^(83)^ Similarly, the majority of informal vendors selling ready-to-eat chicken and its byproducts in Tshwane, South Africa were found to be women who follow good basic hygiene practices. Yet the environments in which they operate were found to be the primary sources of contamination affecting their food.^(84)^ The same applies to the marketing of camel milk in Nairobi.^(85)^ Being located in open areas near drains and roads, and prone to exposure to flooding and dust, dirty water sources, rat infestations and so on means that faecal and environmental contaminants like E. coli and coliforms are likely to find their way through the food chain.^(86)^ Yet female vendors often adopt effective strategies to mitigate microbial risks associated with their products, for instance by preparing ready-to-eat chicken in small amounts at any given time. Such practices reduce the amount of leftover food that needs to be stored or carried over for sale on the following day.^(87)^ Also, Grace et al. found that gendered access to food has been linked to differential exposure to food-borne disease in Ibadan, Nigeria, where women are more likely to consume offal while men enjoy better access to high-value muscle meat.^(88)^ Offal consumption has been found to be a risk factor for diarrhoea.^(89)^

### e. The role of urban planning in disease prevention and mitigation has received scant attention

Urban planners and public health officials worked together, mainly in the 19th and early 20th centuries in US and European cities, to defeat infectious diseases in rapidly changing but unsafe living conditions. Yet we have seldom witnessed similar concerted efforts across much of the urban global South. Beyond such discriminatory approaches as the colonial segregation acts to separate communities from white colonial settlers or administrators on public health grounds,^(90)^ more recent and positive action has largely been limited to the Healthy Cities Movement. Initiated by Trevor Hancock and Leonard Dahl in 1991, the Healthy Cities project was unable to mainstream a health perspective sufficiently into city planning, because of the inadequate financial and institutional support.^(91)^ Moreover, where they exist, current global public health and planning discourses are essentially aligned to non-communicable diseases like some respiratory diseases and those related to obesity. Thus the planning, urban form and design principles they mostly promote tend to advise more active and green spaces, cycling networks, and so on.^(92)^ Infectious diseases, particularly zoonoses, have only rarely been the focus of similar efforts mainstreamed into urban planning and design discourses.

Public health policies and practices are often disconnected from urban planning and development efforts, which allow narratives on the history of settlement formation and inequality, migration, city form and spatial segregation to be overlooked. Meanwhile, historically entrenched differential access to resources or “deep distribution”^(93)^ may have a significant role in creating disease outbreak tipping points through a “mutually reinforcing nexus”. That is, continued public disinvestment leads to the continued decay of social and physical capital and health-enabling elements, particularly within poorer marginalized communities.^(94)^ Interaction and movements of animals, goods and people that often rely on past behaviour and social networks can also be affected by the “lock-in” effects of mega-transport projects^(95)^ that significantly alter agricultural and natural habitats.

For instance, in Nairobi, Kenya’s capital, we found that one of the current drivers to make the city “world class” and more attractive to foreign investors is to introduce modern bypasses and highways to decongest the city and connect the central business district with suburban middle- and high-income residential areas. This rarely caters for the needs of the majority, however, who do not have access to private cars and depend instead on the provision of public transport that seldom uses such infrastructure and is starved of public investment. Coffee farms and other agricultural lands are being converted to residential and commercial land uses along the northern and western road corridors, altering the local livelihood base, while land grabbing and speculation artificially raise land values. According to local stakeholders’ experience and perceptions, urban agriculture, particularly livestock keeping in Nairobi and its periphery, is unable to sustain such disproportionate increases in land value. Landowners thus opt for development instead, and large tracts of formerly agricultural land are turned over to multi-storey buildings, accommodating Nairobi’s “push out” urbanization drives. Livestock keeping survives by shifting to zero-grazing forms, e.g. poultry on plots of decreasing size, as increasingly valuable land is subdivided and sold for more profitable ventures. In consequence, livestock and its material flows (i.e. meat, dairy and poultry) tend to move further away from Nairobi.^(96)^

## V. Concluding Remarks: From Wicked Problems to Transformative Knowledge

From the evidence scan, the direct or indirect role of urbanization drivers in relation to zoonotic diseases can be said to arise from rapid urban growth and increased density of human occupation, increased movement of people and animals, increased complexity in the value chains around animal-sourced products, rural-to-urban migration, intra-city inequalities and land-use changes. Yet we find that epistemological biases can cause scholars to miss other locally grounded drivers at play. Most of the peer-reviewed studies included here were heavily reliant on techno-scientific realist models that often determine disease outcomes based on individual determinants, sidelining “upstream” factors. These factors include wider, structural socioeconomic issues, urban planning issues like the lock-in factor of development (e.g. the introduction of highways and housing in rural or peri-urban agricultural areas and water bodies that are irreversibly converted to urban uses), and the poor living conditions that result in certain places from low productivity levels, lack of public investment and social inequalities. Besides, the insufficient allowance for informality and local, culturally embedded practices within the scholarship allows for perpetuation of politically defined, dominant local policy narratives, dictated by a narrow perception of the risks underpinning such diseases.

As discussed earlier, studies reveal that perceived risks are not always in line with the evidence. For example, chicken rearing is not responsible for diarrhoea in Kampala, and similarly in some contexts, vegetable consumption might carry more risks than the consumption of dairy products from the same systems, as reported by Grace et al., Dimoulas et al. and Alarcón et al.^(97)^ Many hasty reactions from national or local policymakers, favouring large-scale formal enterprises while seeking to limit the action of smaller informal ones, are based on a global narrative that associates unsafe food with informal markets. However, such assumptions are rarely locally contextualized, grounded or tested for evidence. In other words, they are mostly based, in Grace’s words, on a “matter of concern” instead of a “matter of evidence”.^(98)^

As health and equity become substantive issues in the post-2015 development agenda,^(99)^ we need to use the transition platform offered by the Millennium Development Goals (MDGs) and Sustainable Development Goals (SDGs) to recognize the right to health as part of a holistic agenda that goes beyond healthcare access for all. This holistic agenda would include an equitably healthy social and physical environment for all, one that protects marginalized communities more than others.^(100)^ With more people living in urban areas than ever before, the goal of pursuing socially and environmentally just urban development (aiming for social justice and equity, while improving human wellbeing and preserving environmental integrity) is becoming increasingly complex. Responses must take this complexity into account.

## Notes

1. Harpham and Tanner (1995).

2. Among many others, Harpham (1997); Eckert and Kohler (2014); Rocha et al. (2015).

3. Voinovic et al. (2019), page 7.

4. The term “zoonosis” was coined by Rudolph Virchow in 1855 to describe diseases transmitted between vertebrate animals and humans. Greger (2007); Taylor et al. (2001); Woolhouse and Gowtage-Sequeria (2005).

5. UN DESA (2018).

6. Satterthwaite (2010); WHO (2010).

7. Morgan (2009).

8. Delgado and Narrod (2003); Tacoli et al. (2013).

9. Delgado et al. (1999).

10. Sumberg and Thompson (2013).

11. Pearson et al. (2005); Grace, Kang’ethe et al. (2012); FAO (2006).

12. Thornton (2010); FAO (2006).

13. Thornton (2010).

14. Roesel and Grace (2014); Dominguez-Salas et al. (2016).

15. Li et al. (2012); Bai et al. (2012); Goryakin et al. (2017).

16. Goryakin et al. (2017); Smit et al. (2016); Li et al. (2012); Bai et al. (2012).

17. McGranahan and Satterthwaite (2014).

18. Schelling et al. (2007), page 346.

19. Jouve et al. (2010).

20. Taylor et al. (2001).

21. Woolhouse and Gowtage-Sequeria (2005).

22. The term “emerging” includes not only newly appearing zoonotic diseases but also those that are spreading to new territories. Reemerging diseases are those that appear in significantly more virulent forms after a decline or after a long period of low incidence [Alemayehu (2012); Pearson et al. (2005)].

23. DALY, or disability-adjusted life year, is a well-known indicator to calculate the burden placed by disease upon a given population.

24. According to Prasad et al. (2015), fever-causing zoonoses such as brucellosis and trypanosomiasis are often wrongly identified as malaria. See also Grace, Mutua et al. (2012).

25. Havelaar et al. (2015).

26. Urban, peri-urban, urbanisation, urban growth, urban planning, urban development, city, urbanicity, urban health, well-being, urban poverty, slums, informal settlements, vulnerability, contact networks, social networks, social determinants, environmental determinants, drivers, spatial, linkages, exposure, socio-economic, environment, physical environment, built environment, Environmental health, living conditions, disease emergence, re-emergence, infectious diseases, zoonotic diseases, zoonoses, human-animal interface, land-use, disease burden, livestock, food, agriculture, risk.

27. Woolhouse and Gowtage-Sequeria (2005).

28. Mackenstedt et al. (2015), page 71.

29. Allen et al. (2017).

30. Rydin et al. (2012).

31. Moore et al. (2003).

32. Sverdlik (2011).

33. UN-Habitat (2010).

34. Satterthwaite (2016).

35. Alirol et al. (2011).

36. Maciel et al. (2008); Sarkar et al. (2002).

37. Reis et al. (2008).

38. Patel et al. (2009).

39. Dimoulas et al. (2008).

40. Katukiza et al. (2014).

41. Katukiza et al. (2014), page 455.

42. Alirol et al. (2011).

43. Reyburn et al. (2003).

44. Tacoli (1998); Allen et al. (2015).

45. Alarcón, Fèvre, Murungi et al. (2017).

46. Leach and Scoones (2013); Slingenbergh et al. (2004).

47. FAO (2007).

48. Abdo-Salem et al. (2011).

49. Munyua et al. (2010).

50. Kiambi et al. (2018).

51. Alarcón, Fèvre, Muindi et al. (2017).

52. Woolhouse and Gowtage-Sequeria (2005).

53. Satterthwaite (2010); UN-Habitat (2009).

54. Dewan and Yamaguchi (2009).

55. Alirol et al. (2011).

56. McFarlane et al. (2013).

57. McFarlane et al. (2013).

58. Antimicrobial resistance is the ability of a microbe to resist the effects of a drug that once might have successfully eradicated the microbe. This is a growing problem around the world, partly linked to increased and indiscriminate consumption of antibiotics.

59. Hassell, Ward, Muloi, Bettridge, Robinson, Ogendo et al. (2019); Hassell, Ward, Muloi, Bettridge, Robinson, Kariuki et al. (2019).

60. Leach et al. (2010a).

61. van Woezik et al. (2016), page 2.

62. Bai et al. (2012), page 438.

63. Bai et al. (2012), page 465.

64. Gatrell (2005), page 2662.

65. Gatrell (2005).

66. See for example Leach et al. (2010b); Stirling and Scoones (2009); and a special issue in the *Philosophical Transactions of the Royal Society B* edited by Cunningham et al. (2017).

67. Craddock and Hinchliffe (2015), page 2.

68. Dzingirai et al. (2017), pages 4–5.

69. Wallensteen and Sollenberg (2001).

70. Alarcón, Fèvre, Muindi et al. (2017).

71. Grace, Kang’ethe et al. (2012); Grace, Randolph et al. (2012).

72. Mdegela et al. (2006).

73. Grace (2014), page 2.

74. Roesel and Grace (2014).

75. Grace, Nasinyama et al. (2008); Grace, Omore et al. (2008).

76. Kang’ethe et al. (2012); McDermott et al. (2012).

77. Grace, Kang’ethe et al. (2012); Grace, Randolph et al. (2012); Ahmed et al. (2015); Ahmed et al. (2019); Muloi et al. (2018).

78. Grace (2014), page 2.

79. Dalziel et al. (2013).

80. Grace et al. (2015).

81. Kang’ethe et al. (2012); McDermott et al. (2012); Kimani et al. (2012).

82. Kang’ethe, et al. (2012); McDermott et al. (2012).

83. Grace (2014), page 3; Roesel and Grace (2014).

84. Oguttu et al. (2015).

85. Muloi et al. (2018).

86. Oguttu et al. (2015).

87. Oguttu et al. (2015).

88. Grace, Olowoye et al. (2012).

89. Grace, Olowoye et al. (2012).

90. Njoh (2012).

91. Corburn (2009); Harpham et al. (2001).

92. For examples of city planning processes and human health impacts, see Table 3.2 in Corburn (2013), page 71.

93. As described by Levy (2013).

94. Wallace and Wallace (1997), page 798.

95. Watson (2014).

96. Ahmed and Dávila (2016), page 53.

97. Grace, Kang’ethe et al. (2012); Dimoulas et al. (2008); Alarcón, Fèvre, Murungi et al. (2017).

98. Grace (2014).

99. Ruano et al. (2014).

100. Ooms et al. (2013).

## References

[bibr1-0956247819866124] Abdo-Salem S Tran A Grosbois V Gerbier G Al-Qadasi M Saeed K Etter E Thiry E Roger F Chevalier V (2011), “Can environmental and socioeconomic factors explain the recent emergence of Rift Valley fever in Yemen, 2000–2001?”, Vector Borne and Zoonotic Diseases Vol 11, No 6, pages 773–779. 10.1089/vbz.2010.0084 21284504

[bibr2-0956247819866124] Ahmed S Dávila J D (2015), “Institutional mapping of Nairobi: a critical look into current policies and institutions in the context of land, land-use and infrastructure provision”, unpublished manuscript.

[bibr3-0956247819866124] Ahmed S Haklay M Tacoli C Githiri G Dávila J Allen A Fèvre E (2019), “Participatory mapping and food-centred justice in informal settlements in Nairobi, Kenya”, Geo: Geography and Environment Vol 6, No 1.

[bibr4-0956247819866124] Ahmed S Simiyu E Githiri G Sverdlik A Mbaka S (2015), “Cooking up a storm: community-led mapping and advocacy with food vendors in Nairobi’s informal settlements”, working paper, International Institute for Environment and Development, London.

[bibr5-0956247819866124] Alarcón P Fèvre E Muinde P Murungi M K Kiambi S Akoko J Rushton J (2017), “Urban livestock keeping in the city of Nairobi: diversity of production systems, supply chains, and their disease management and risks”, Frontiers in Veterinary Science Vol 4, No 171. 10.3389/fvets.2017.00171 PMC5669286 29164137

[bibr6-0956247819866124] Alarcón P Fèvre E M Murungi M K Muinde P Akoko J Dominguez-Salas P Kiambi S Ahmed S Häsler B Rushton J (2017), “Mapping of beef, sheep and goat food systems in Nairobi - a framework for policy making and the identification of structural vulnerabilities and deficiencies”, Agricultural Systems Vol 152, pages 1–17. 10.1016/j.agsy.2016.12.005 PMC5312657 28260829

[bibr7-0956247819866124] Alemayehu A (2012), “Review on emerging and re-emerging bacterial zoonotic diseases”, American-Eurasian Journal of Scientific Research Vol 7, No 4, pages 176–186.

[bibr8-0956247819866124] Alirol E Getaz L Stoll B Chappuis F Loutan L (2011), “Urbanisation and infectious diseases in a globalised world”, The Lancet Infectious Diseases Vol 11, No 2, pages 131–141. 10.1016/S1473-3099(10)70223-1 PMC7106397 21272793

[bibr9-0956247819866124] Allen A Brown D Dávila J D Hofmann P (2015), Topic Guide: Building Reciprocal Rural-Urban Linkages through Infrastructure Investment and Development, Evidence on Demand.

[bibr10-0956247819866124] Allen T Murray K A Zambrana-Torrelio C Morse S S Rondinini C Di Marco M Breit N Olival K J Daszak P (2017), “Global hotspots and correlates of emerging zoonotic diseases”, Nature Communications Vol 8, No 1. 10.1038/s41467-017-00923-8 PMC5654761 29066781

[bibr11-0956247819866124] Bai X Nath I Capon A Hasan N Jaron D (2012), “Health and wellbeing in the changing urban environment: complex challenges, scientific responses, and the way forward”, Current Opinion in Environmental Sustainability Vol 4, No 4, pages 465–472.

[bibr12-0956247819866124] Corburn J (2009), Toward the healthy city: people, places, and the politics of urban planning, MIT Press.

[bibr13-0956247819866124] Corburn J (2013), “Collaborative planning in Nairobi’s slums”, in Healthy City Planning: From Neighbourhood to National Health Equity, Routledge, London, pages 103–129.

[bibr14-0956247819866124] Craddock S Hinchliffe S (2015), “One world, one health? Social science engagement with the one health agenda”, Social Science & Medicine Vol 129, pages 1–4. 10.1016/j.socscimed.2014.11.016 25434985

[bibr15-0956247819866124] Cunningham A A Scoones I Wood J L (2017), “One Health for a changing world: new perspectives from Africa”, Philosophical Transactions of the Royal Society B: Biological Sciences Vol 372, No 1725, pages 1–7. 10.1098/rstb.2016.0162 PMC5468687 28584170

[bibr16-0956247819866124] Dalziel B D Pourbohloul B Ellner S P (2013), “Human mobility patterns predict divergent epidemic dynamics among cities”, Proceedings of the Royal Society B: Biological Sciences Vol 280, No 1766. 10.1098/rspb.2013.0763 PMC3730584 23864593

[bibr17-0956247819866124] Delgado C L Narrod C A (2003), Policy, Technical, and Environmental Determinants and Implications of the Scaling-up of Livestock Production in Four Fast-Growing Developing Countries: A Synthesis, International Food Policy Research Institute.

[bibr18-0956247819866124] Delgado C Rosegrant M Steinfeld H Ehui S Courbois C (1999), “Livestock to 2020: the next food revolution”, Food, Agriculture, and the Environment Discussion Paper 28, International Food Policy Research Institute, Washington, DC.

[bibr19-0956247819866124] Dewan A M Yamaguchi Y (2009), “Land use and land cover change in Greater Dhaka, Bangladesh: using remote sensing to promote sustainable urbanization”, Applied Geography Vol 29, No 3, pages 390–401.

[bibr20-0956247819866124] Dimoulas P Waltner-Toews D Humphries S Nainyama G W (2008), “Household risk factors associated with chicken rearing and food consumption in Kampala”, in Cole D C Lee-Smith D Nasinyama G W (editors), Healthy City Harvests: Generating Evidence to Guide Policy on Urban Agriculture, CIP/Urban Harvest and Makerere University Press, Kampala and Lima, pages 177–192.

[bibr21-0956247819866124] Dominguez-Salas P Alarcón P Häsler B Dohoo I R Colverson K Kimani-Murage E W Alonso S Ferguson E Fèvre E M Rushton J Grace D (2016), “Nutritional characterisation of low-income households of Nairobi: socioeconomic, livestock and gender considerations and predictors of malnutrition from a cross-sectional survey”, BMC Nutrition Vol 2.

[bibr22-0956247819866124] Dzingirai V Bukachi S Leach M Mangwanya L Scoones I Wilkinson A (2017), “Structural drivers of vulnerability to zoonotic disease in Africa” Philosophical Transactions of the Royal Society B: Biological Sciences Vol 372, No 1725. 10.1098/rstb.2016.0169 PMC5468694 28584177

[bibr23-0956247819866124] Eckert S Kohler S (2014), “Urbanization and health in developing countries: a systematic review”, World Health & Population Vol 15, No 1, pages 7–20. 10.12927/whp.2014.23722 24702762

[bibr24-0956247819866124] FAO (2006), World Agriculture: Towards 2030/2050, interim report, Global Perspective Studies Unit, Food and Agriculture Organization of the United Nations, Rome.

[bibr25-0956247819866124] FAO (2007), Avian Influenza—Questions & Answers: The Facts of Bird Flu, Agriculture Department, Animal Production and Health Division, Food and Agriculture Organization of the United Nations.

[bibr26-0956247819866124] Gatrell A C (2005), “Complexity theory and geographies of health: a critical assessment”, Social Science & Medicine Vol 60, No 12, pages 2661–2671. 10.1016/j.socscimed.2004.11.002 PMC7131797 15820578

[bibr27-0956247819866124] Goryakin Y Rocco L Suhrcke M (2017), “The contribution of urbanisation to non-communicable diseases: evidence from 173 countries from 1980 to 2008”, Economics & Human Biology Vol 26, pages 151–163. 10.1016/j.ehb.2017.03.004 28410489

[bibr28-0956247819866124] Grace D (2014), “Safe and fair food for informal markets: a food safety impact narrative”, research brief, International Livestock Research Institute, Nairobi.

[bibr29-0956247819866124] Grace D Gilbert J Randolph T Kang’ethe E (2012), “The multiple burdens of zoonotic disease and an ecohealth approach to their assessment”, Tropical Animal Health and Production Vol 44, No 1, pages 67–73. 10.1007/s11250-012-0209-y 22886445

[bibr30-0956247819866124] Grace D Kang’ethe E Waltner-Toews D (2012), “Participatory and integrative approaches to food safety in developing country cities”, Tropical Animal Health and Production Vol 44, Suppl 1, pages 1–2. 10.1007/s11250-012-0200-7 22890480

[bibr31-0956247819866124] Grace D Mutua F Ochungo P (2012), Mapping of Poverty and Likely Zoonoses Hotspots, International Livestock Research Institute, Nairobi, 119 pages.

[bibr32-0956247819866124] Grace D Nasinyama G W Randolph T F Mwiine F Kang’ethe E (2008), “City dairying in Kampala: integrating benefits and harms”, in Cole D C Lee-Smith D Nasinyama G W (editors), Healthy City Harvests: Generating Evidence to Guide Policy on Urban Agriculture, CIP/Urban Harvest and Makerere University Press, Kampala and Lima, pages 193–210.

[bibr33-0956247819866124] Grace D Olowoye J Dipeolu M Odebode S Randolph T (2012), “The influence of gender and group membership on food safety: the case of meat sellers in Bodija market, Ibadan, Nigeria”, Tropical Animal Health and Production Vol 44, No 1, pages 53–59. 10.1007/s11250-012-0207-0 22872520

[bibr34-0956247819866124] Grace D Omore A Randolph T Kang’ethe E Nasinyama G W Mohammed H O (2008), “Risk assessment for Escherichia coli O157:H7 in marketed unpasteurized milk in selected East African countries”, Journal of Food Protection Vol 71, pages 257–263. 10.4315/0362-028x-71.2.257 18326173

[bibr35-0956247819866124] Grace D Roesel K Kabui K (2014), “Gender aspects of informal markets for animal-sourced food”, in Roesel K Grace D (editors), Food Safety and Informal Markets: Animal Products in Sub-Saharan Africa, Routledge, pages 114–124.

[bibr36-0956247819866124] Greger M (2007), “The human/animal interface: emergence and resurgence of zoonotic infectious diseases”, Critical Reviews in Microbiology Vol 33, No 4, pages 243–299. 10.1080/10408410701647594 18033595

[bibr37-0956247819866124] Harpham T (1997), “Urbanization and health in transition”, Lancet Vol 349, Suppl III, pages 11–13.

[bibr38-0956247819866124] Harpham T Burton S Blue I (2001), “Healthy city projects in developing countries: first evaluation”, Health Promotion International Vol 16, No 2, pages 111–125. 10.1093/heapro/16.2.111 11356750

[bibr39-0956247819866124] Harpham T Tanner M (1995), Urban Health in Developing Countries: Progress and Prospects, Routledge.

[bibr40-0956247819866124] Hassell J M Ward M J Muloi D Bettridge J M Robinson T P Ogendo A Imboma T Kiiru J Kariuki S Begon M Kang’ethe E K Woolhouse M E J Fèvre E M (2019), “Deterministic processes structure bacterial genetic communities across an urban landscape”, Nature Communications Vol 10. 10.1038/s41467-019-10595-1 PMC6572833 31201324

[bibr41-0956247819866124] Hassell J M Ward M J Muloi D Bettridge J M Robinson T P Kariuki S Ogendo A Kirru J Imboma T Kang’ethe E Öghren E M Williams N J Begon M Woolhouse M E J Fèvre E M (2019), “Clinically relevant antimicrobial resistance at the wildlife–livestock–human interface in Nairobi: an epidemiological study”, Lancet Planetary Health Vol 3, No 6, pages e259–269. 10.1016/S2542-5196(19)30083-X PMC6630895 31229001

[bibr42-0956247819866124] Havelaar A H Kirk M D Torgerson P R Gibb H J Hald T Lake R J Praet N Bellinger D C de Silva N R Gargouri N Speybroeck N Cawthorne A Mathers C Stein C Angulo F J Devleesschauwer B (2015), “World Health Organization global estimates and regional comparisons of the burden of foodborne disease in 2010”, PLOS Medicine Vol 12, No 12. 10.1371/journal.pmed.1001923 PMC4668832 26633896

[bibr43-0956247819866124] Jouve J-L Aagaard-Hansen J Aidara-Kane A (2010), “Food safety: equity and social determinants”, in Blas E Sivasankara Kurup A (editors), Equity, Social Determinants and Public Health Programmes, World Health Organization, Geneva, pages 95–114.

[bibr44-0956247819866124] Kang’ethe E K Kimani V N McDermott B Grace D Lang’at A K Kiragu M W Karanja N Njehu A N Randolph T Mbugua G Irungu T W Ombutu P (2012), “A trans-disciplinary study on the health risks of cryptosporidiosis from dairy systems in Dagoretti, Nairobi, Kenya: study background and farming system characteristics”, Tropical Animal Health and Production Vol 44, No 1, pages 3–10. 10.1007/s11250-012-0199-9 22886442

[bibr45-0956247819866124] Katukiza A Y Ronteltap M Steen P Foppen J W A Lens P N L (2014), “Quantification of microbial risks to human health caused by waterborne viruses and bacteria in an urban slum”, Journal of Applied Microbiology Vol 116, No 2, pages 447–463. 10.1111/jam.12368 24127653

[bibr46-0956247819866124] Kiambi S Alarcón P Rushton J Murungi M K Muinde P Akoko J Aboge G Gikonyo S Momanyi K Kang’ethe E K Fèvre E (2018), “Mapping Nairobi’s dairy food system: an essential analysis for industry and research”, Agricultural Systems Vol 167, pages 47–60. 10.1016/j.agsy.2018.08.007 PMC6358146 30739979

[bibr47-0956247819866124] Kimani V N Mitoko G McDermott B Grace D Ambia J Kiragu M W Njehu A N Sinja J Monda J G Kang’ethe E K (2012), “Social and gender determinants of risk of cryptosporidiosis, an emerging zoonosis, in Dagoretti, Nairobi, Kenya”, Tropical Animal Health and Production Vol 44, No 1, pages 17–23. 10.1007/s11250-012-0203-4 22865349

[bibr48-0956247819866124] Leach M Scoones I (2013), “The social and political lives of zoonotic disease models: narratives, science and policy”, Social Science & Medicine Vol 88, pages 10–17. 10.1016/j.socscimed.2013.03.017 23702205

[bibr49-0956247819866124] Leach M Scoones I Stirling A (2010a), “Governing epidemics in an age of complexity: narratives, politics and pathways to sustainability”, Global Environmental Change Vol 20, No 3, pages 369–377.

[bibr50-0956247819866124] Leach M Scoones I Stirling A (2010b), Dynamic Sustainabilities: Technology, Environment, Social Justice, Earthscan.

[bibr51-0956247819866124] Levy C (2013), “Travel choice reframed: “deep distribution” and gender in urban transport”, Environment and Urbanization Vol 25, No 1, pages 47–63.

[bibr52-0956247819866124] Li X H Liu J L Gibson V Zhu Y G (2012), “Urban sustainability and human health in China, East Asia and Southeast Asia”, Current Opinion in Environmental Sustainability Vol 4, No 4, pages 436–442.

[bibr53-0956247819866124] MacGregor H Waldman L (2017), “Views from many worlds: unsettling categories in interdisciplinary research on endemic zoonotic diseases”, Philosophical Transactions of the Royal Society B: Biological Sciences Vol 372, No 1725. 10.1098/rstb.2016.0170 PMC5468695 28584178

[bibr54-0956247819866124] Maciel E A de Carvalho A L F Nascimento S F de Matos R B Gouveia E L Reis M G Ko A I (2008), “Household transmission of Leptospira infection in urban slum communities”, PLOS Neglected Tropical Diseases Vol 2, No 1. 10.1371/journal.pntd.0000154 PMC2270796 18357340

[bibr55-0956247819866124] Mackenstedt U Jenkins D Romig T (2015), “The role of wildlife in the transmission of parasitic zoonoses in peri-urban and urban areas”, International Journal for Parasitology: Parasites and Wildlife Vol 4, No 1, pages 71–79. 10.1016/j.ijppaw.2015.01.006 PMC4356871 25830108

[bibr56-0956247819866124] McDermott B Grace D Mbae C Mulinge E Monda J Nyongesa C Ambia J Njehu A (2012), “Prevalence of cryptosporidiosis in dairy cattle, cattle-keeping families, their non-cattle-keeping neighbours and HIV-positive individuals in Dagoretti Division, Nairobi, Kenya”, Tropical Animal Health and Production Vol 44, No 1, pages 11–16. 10.1007/s11250-012-0201-6 22878888

[bibr57-0956247819866124] McFarlane R A Sleigh A C McMichael A J (2013), “Land-use change and emerging infectious disease on an island continent”, International Journal of Environmental Research and Public Health Vol 10, No 7, pages 2699–2719. 10.3390/ijerph10072699 PMC3734451 23812027

[bibr58-0956247819866124] McGranahan G Satterthwaite D (2014), “Urbanisation: concepts and trends”, working paper, International Institute for Environment and Development, London.

[bibr59-0956247819866124] Mdegela R H Nonga H E Ngowi H A Kazwala R R (2006), “Prevalence of thermophilic campylobacter infections in humans, chickens and crows in Morogoro, Tanzania”, Journal of Veterinary Medicine B: Infectious Diseases and Veterinary Public Health Vol 53, No 3, 116–121. 10.1111/j.1439-0450.2006.00926.x 16629722

[bibr60-0956247819866124] Moore M Gould P Keary B S (2003), “Global urbanization and impact on health”, International Journal of Hygiene and Environmental Health Vol 206, pages 269–278. 10.1078/1438-4639-00223 12971682

[bibr61-0956247819866124] Morgan K (2009), “Feeding the city: the challenge of urban food planning”, International Planning Studies Vol 14, No 4, pages 341–348.

[bibr62-0956247819866124] Muloi D Alarcón P Ombui J Ngeiywa K J Abdullahi B Muinde P Karani M K Rushton J Fèvre E M (2018), “Value chain analysis and sanitary risks of the camel milk system supplying Nairobi city, Kenya”, Preventive Veterinary Medicine Vol 159, pages 203–210. 10.1016/j.prevetmed.2018.09.010 PMC6193137 30314783

[bibr63-0956247819866124] Munyua P Murithi R M Wainwright S Githinji J Hightower A Mutonga D Macharia J Ithondeka P M Mussaa J Breiman R F Bloland P Njenga M K (2010), “Rift Valley fever outbreak in livestock in Kenya, 2006–2007”, The American Journal of Tropical Medicine and Hygiene Vol 83, No 2, Suppl, pages 58–64. 10.4269/ajtmh.2010.09-0292 PMC2913503 20682907

[bibr64-0956247819866124] Njoh A (2012), Urban Planning and Public Health in Africa, Ashgate.

[bibr65-0956247819866124] Oguttu J Roesel K McCrindle C Hendrickx S Makita K Grace D (2015), “Arrive alive in South Africa: chicken meat the least to worry about”, in Roesel K Grace D (editors), Food Safety and Informal Markets: Animal Products in Sub-Saharan Africa, Routledge, pages 202–220.

[bibr66-0956247819866124] Ooms G Brolan C Eggermont N Eide A Flores W Forman L Friedman E A Gebauer T Gostin L O Hill P S Hussain S McKee M Mulumba M Siddiqui F Sridhar D Van Leemput L Waris A Jahn A (2013), “Universal health coverage anchored in the right to health”, Bulletin of the World Health Organization Vol 91, No 1. 10.2471/BLT.12.115808 PMC3537254 23397341

[bibr67-0956247819866124] Patel N T Patel N H Madan L T (2009), “Outbreak of urban leptospirosis in Surat post floods”, Gujarat Medical Journal Vol 64, No 1, pages 39–41.

[bibr68-0956247819866124] Pearson J Salman M D BenJabara K Brown C Formenty P Griot C James A Jemmi T King L Lautner E McCluskey B J Meslin F X Ragan V (2005), “Global risks of infectious animal diseases”, Issue Paper No 28, Council for Agricultural Science and Technology.

[bibr69-0956247819866124] Prasad N Murdoch D R Reyburn H Crump J A (2015), “Etiology of severe febrile illness in low-and middle-income countries: a systematic review”, PLOS ONE Vol 10, No 6. 10.1371/journal.pone.0127962 PMC4488327 26126200

[bibr70-0956247819866124] Reis R B Ribeiro G S Felzemburgh R D M Santana F S Mohr S Melendez A X T O Queiroz A Santos A C Ravines R R Tassinari W S Carvalho M S Reis M G Ko A I (2008), “Impact of environment and social gradient on leptospira infection in urban slums”, PLoS Neglected Tropical Diseases Vol 2, No 4. 10.1371/journal.pntd.0000228 PMC2292260 18431445

[bibr71-0956247819866124] Reyburn H Rowland M Mohsen M Khan B Davies C (2003), “The prolonged epidemic of anthroponotic cutaneous leishmaniasis in Kabul, Afghanistan: ‘bringing down the neighbourhood’”, Transactions of the Royal Society of Tropical Medicine and Hygiene Vol 97, pages 170–176. 10.1016/s0035-9203(03)90111-8 14584372

[bibr72-0956247819866124] Rocha L E Thorson A E Lambiotte R (2015), “The non-linear health consequences of living in larger cities”, Journal of Urban Health Vol 92, No 5, pages 785–799. 10.1007/s11524-015-9976-x PMC4608943 26245466

[bibr73-0956247819866124] Roesel K Grace D (editors) (2014), Food Safety and Informal Markets: Animal Products in Sub-Saharan Africa, Routledge, London.

[bibr74-0956247819866124] Ruano A L Friedman E A Hill P S (2014), “Health, equity and the post-2015 agenda: raising the voices of marginalized communities”, International Journal for Equity in Health Vol 13, No 1. 10.1186/s12939-014-0082-6 PMC4201725 25300905

[bibr75-0956247819866124] Rydin Y Bleahu A Davies M Dávila J D Friel S De Grandis G Groce Nora Hallal P C Hamilton I Howden-Chapman P Lai K-M Lim C J Martins J Osrin D Ridley I Scott I Taylor M Wilkinson P Wilson J (2012), “Shaping cities for health: complexity and the planning of urban environments in the 21st century”, Lancet Vol 379, No 9831, pages 2079–2108. 10.1016/S0140-6736(12)60435-8 PMC3428861 22651973

[bibr76-0956247819866124] Sarkar U Nascimento S F Barbosa R Martins R Nuevo H Kalofonos I Grunstein I Flannery B Dias J Riley L W Reis M G Ko A I (2002), “Population-based case-control investigation of risk factors for leptospirosis during an urban epidemic”, American Journal of Tropical Medicine and Hygiene Vol 66, No 5, pages 605–610. 10.4269/ajtmh.2002.66.605 12201599

[bibr77-0956247819866124] Satterthwaite D (2010), “Urban myths and the mis-use of data that underpin them”, Working paper 2228, World Institute for Development Economics Research.

[bibr78-0956247819866124] Satterthwaite D (2016), “Missing the Millennium Development Goal targets for water and sanitation in urban areas”, Environment and Urbanization Vol 28, No 1, pages 99–118.

[bibr79-0956247819866124] Schelling E Grace D Willingham A L III Randolph T (2007), “Research approaches for improved pro-poor control of zoonoses”, Food and Nutrition Bulletin Vol 28, No 2, Suppl 2, pages S345–S356. 10.1177/15648265070282S214 17658081

[bibr80-0956247819866124] Slingenbergh J I Gilbert M de Balogh K I Wint W (2004), “Ecological sources of zoonotic diseases”, Scientific and Technical Review of the Office International des Epizooties Vol 23, pages 467–484. 10.20506/rst.23.2.1492 15702714

[bibr81-0956247819866124] Smit W De Lannoy A Dover R V Lambert E V Levitt N Watson V (2016), “Making unhealthy places: the built environment and non-communicable diseases in Khayelitsha, Cape Town”, Health & Place Vol 39, pages 196–203. 10.1016/j.healthplace.2016.04.006 27157313

[bibr82-0956247819866124] Stirling A C Scoones I (2009), “From risk assessment to knowledge mapping: science, precaution, and participation in disease ecology”, Ecology and Society Vol 14, No 2.

[bibr83-0956247819866124] Sumberg J Thompson J (2013), “Revolution reconsidered: evolving perspectives on livestock production and consumption”, STEPS Working Paper 52, STEPS Centre, Brighton.

[bibr84-0956247819866124] Sverdlik A (2011), “Ill-health and poverty: a literature review on health in informal settlements”, Environment and Urbanization Vol 23, No 1, pages 123–155.

[bibr85-0956247819866124] Tacoli C (1998), “Rural-urban interactions: a guide to the literature”, Environment and Urbanization Vol 10, No 1, pages 147–166.

[bibr86-0956247819866124] Tacoli C Bukhari B Fisher S (2013), “Urban poverty, food security and climate change”, Human Settlements Working Paper 37, International Institute for Environment and Development, London.

[bibr87-0956247819866124] Taylor L H Latham S M Woolhouse M E J (2001), “Risk factors for human disease emergence”, Philosophical Transactions of the Royal Society of London B: Biological Sciences Vol 356, No 1411, pages 983–989. 10.1098/rstb.2001.0888 PMC1088493 11516376

[bibr88-0956247819866124] Thornton P K (2010), “Livestock production: recent trends, future prospects”, Philosophical Transactions of the Royal Society of London B: Biological Sciences Vol 365, No 1554, pages 2853–2867. 10.1098/rstb.2010.0134 PMC2935116 20713389

[bibr89-0956247819866124] UN DESA (2018), Promoting Inclusion through Social Protection: Report on the World Social Situation 2018, United Nations Department of Economic and Social Affairs.

[bibr90-0956247819866124] UN-Habitat (2009), Planning Sustainable Cities: Global Report on Human Settlements 2009, Earthscan and United Nations Human Settlements Programme, London.

[bibr91-0956247819866124] UN-Habitat (2010), State of African Cities 2010: Governance, Inequalities and Urban Land Markets, Nairobi, 277 pages.

[bibr92-0956247819866124] van Woezik A F Braakman-Jansen L M Kulyk O Siemons L van Gemert-Pijnen J E (2016), “Tackling wicked problems in infection prevention and control: a guideline for co-creation with stakeholders”, Antimicrobial Resistance & Infection Control Vol 5, No 1. 10.1186/s13756-016-0119-2 PMC4875594 27213040

[bibr93-0956247819866124] Vojnovic I Pearson A L Asiki G DeVerteuil G Allen A (editors) (2019), Handbook of Global Urban Health, Routledge, New York and London.

[bibr94-0956247819866124] Wallace R Wallace D (1997), “Resilience and persistence of the synergism of plagues: stochastic resonance and the ecology of disease, disorder and disinvestment in US urban neighborhoods”, Environment and Planning A Vol 29, No 5, pages 789–804.

[bibr95-0956247819866124] Wallensteen P Sollenberg M (2001), “Armed conflict, 1989–2000”, Journal of Peace Research Vol 38, No 5, pages 629–644.

[bibr96-0956247819866124] Watson V (2014), “African urban fantasies: dreams or nightmares?”, Environment and Urbanization Vol 26, No 1, pages 215–231.

[bibr97-0956247819866124] WHO (2010), Unmasking and Overcoming Health Inequities in Urban Settings, World Health Organization, Geneva.

[bibr98-0956247819866124] Woolhouse M E J Gowtage-Sequeria S (2005), “Host range and emerging and reemerging pathogens”, Emerging Infectious Diseases Vol 11, No 12, pages 1842–1847. 10.3201/eid1112.050997 PMC3367654 16485468

